# Characterisation of a cyclic peptide that binds to the RAS binding domain of phosphoinositide 3-kinase p110α

**DOI:** 10.1038/s41598-023-28756-0

**Published:** 2023-02-02

**Authors:** Mohamed Ismail, Stephen R. Martin, Roger George, Francesca Houghton, Geoff Kelly, Raphaël A. G. Chaleil, Panayiotis Anastasiou, Xinyue Wang, Nicola O’Reilly, Stefania Federico, Dhira Joshi, Hemavathi Nagaraj, Rachel Cooley, Ning Sze Hui, Miriam Molina-Arcas, David C. Hancock, Ali Tavassoli, Julian Downward

**Affiliations:** 1 Oncogene Biology Laboratory, Francis Crick Institute, 1 Midland Road, London, NW1 1AT UK; 2 Structural Biology, Science Technology Platforms, Francis Crick Institute, 1 Midland Road, London, NW1 1AT UK; 3 Peptide Chemistry, Science Technology Platforms, Francis Crick Institute, 1 Midland Road, London, NW1 1AT UK; 4 Biomolecular Modelling Lab, Francis Crick Institute, 1 Midland Road, London, NW1 1AT UK; 5 School of Chemistry, University of Southampton, Southampton, SO17 1BJ UK

**Keywords:** Oncogenes, Cancer, Drug discovery, Drug screening

## Abstract

P110α is a member of the phosphoinositide 3-kinase (PI3K) enzyme family that functions downstream of RAS. RAS proteins contribute to the activation of p110α by interacting directly with its RAS binding domain (RBD), resulting in the promotion of many cellular functions such as cell growth, proliferation and survival. Previous work from our lab has highlighted the importance of the p110α/RAS interaction in tumour initiation and growth. Here we report the discovery and characterisation of a cyclic peptide inhibitor (cyclo-CRVLIR) that interacts with the p110α-RBD and blocks its interaction with KRAS. cyclo-CRVLIR was discovered by screening a “split-intein cyclisation of peptides and proteins” (SICLOPPS) cyclic peptide library. The primary cyclic peptide hit from the screen initially showed a weak affinity for the p110α-RBD (K_d_ about 360 µM). However, two rounds of amino acid substitution led to cyclo-CRVLIR, with an improved affinity for p110α-RBD in the low µM (K_d_ 3 µM). We show that cyclo-CRVLIR binds selectively to the p110α-RBD but not to KRAS or the structurally-related RAF-RBD. Further, using biophysical, biochemical and cellular assays, we show that cyclo-CRVLIR effectively blocks the p110α/KRAS interaction in a dose dependent manner and reduces phospho-AKT levels in several oncogenic KRAS cell lines.

## Introduction

PI3K is a lipid kinase family that phosphorylates the 3′ position of the inositol group of phosphoinositide (PtdIns) lipids, converting PtdIns(4,5)P_2_ to PtdIns(3,4,5)P_3_, which serves as a functional regulator of a wide network of proteins through its binding to pleckstrin homology domains. There are 3 classes of PI3K: Class I, which function to transduce signals arising from plasma membrane-bound receptors and small GTPases, and Class II and III, that are involved in membrane and vesicle trafficking^1–3^. Class I PI3K function as heterodimers comprised of a catalytic p110 subunit in complex a regulatory subunit, with class 1A enzymes (p110α, p110β and p110δ) associating with the SH2 domain containing regulatory subunits (p85α, p85β or p55γ), and class 1B p110γ binding to a p101 regulatory subunit^1,4,5^. All Class I PI3K p110 subunits contain a RAS binding domain (RBD) that is involved in the interaction with small GTPases. It has previously been shown that RAS GTPases interact with p110α, p110 δ and p110γ, whereas p110β interacts with RAC1 and CDC42^6,7^.

RAS proteins are members of the GTPase superfamily of proteins and consist of four isoforms: HRAS, NRAS, KRAS4A and KRAS4B. RAS proteins behave as molecular switches, oscillating between an active RAS-GTP and an inactive RAS-GDP state. The exchange between GTP- and GDP-bound states structurally alters two regions of RAS named switch I (residues 28–38) and switch II (57–63)^8,9^. RAS-GTP promotes effector binding and activates downstream signalling, most notably the PI3K and RAF pathways, involved in the control of various cellular functions such as growth, survival, differentiation and proliferation. RAS oncogenic mutations are found in some 20% of human cancers. KRAS is the most mutated protein in the RAS family representing almost 85% of RAS oncogenic mutations, in comparison with NRAS 12% and HRAS 3%. RAS oncogenic mutations occur mainly at amino acids G12, G13 or Q61. These mutations prevent the hydrolysis of GTP to GDP resulting in a constantly active state of RAS that persistently activates downstream signalling pathways thereby contributing to the genesis and maintenance of several types of cancer^10,11^. Over the past thirty years there have been numerous efforts to find RAS inhibitors, however, until very recently these were unsuccessful^12–16^. This has changed in the past few years, with effective RAS inhibitors, such as AMG 510 (sotorasib), MRTX849 (adagrasib) and ARS-1620, being made that directly target a specific oncogenic mutant residue in KRAS, G12C. However, although these drugs hold much promise for the treatment of tumours with this specific mutation, they are only suitable for around 14% of total RAS oncogenic mutant tumours^17–19^.

Previous work from our laboratory showed that it is possible to block the p110α/RAS interaction by introducing genetic mutations to the key p110α-RBD residues, T208D and K227A. Introduction of these mutations into mice carrying RAS oncogenes led to strong resistance to tumour growth, initiation and maintenance. Furthermore, blocking the p110α/RAS interaction in wild type mice was found to be well tolerated, which highlights the significance of the p110α/RAS interaction as a cancer drug target^20–22^. Therefore, we were interested in finding inhibitors that block the interaction of p110α with RAS for future pharmaceutical studies.

We used a SICLOPPS library for the production and screening of cyclic peptides in bacteria to identify an inhibitor of the p110α/RAS interaction^23–29^. Cyclic peptides have several advantages over small molecules, including their greater size, which can enhance their capability for blocking protein–protein interactions (PPI). The N-to-C terminal cyclisation gives a rigid structure, which maintains the cyclic peptide conformation and reduces cellular hydrolysis. In addition, peptides in general are known to have relatively low toxicity^30,31^. Through several rounds of peptide screening and optimisation, we report a 6 amino acid unit containing cyclic peptide (CRVLIR) named cyclo-CRVLIR that is capable of blocking the p110α/RAS interaction in vitro and in several KRAS cancer cell lines.

## Results

### Use of SICLOPPS to identify a cyclo-CRVLAA peptide that inhibits the p110α-RBD/KRAS interaction

**Figure 1:**
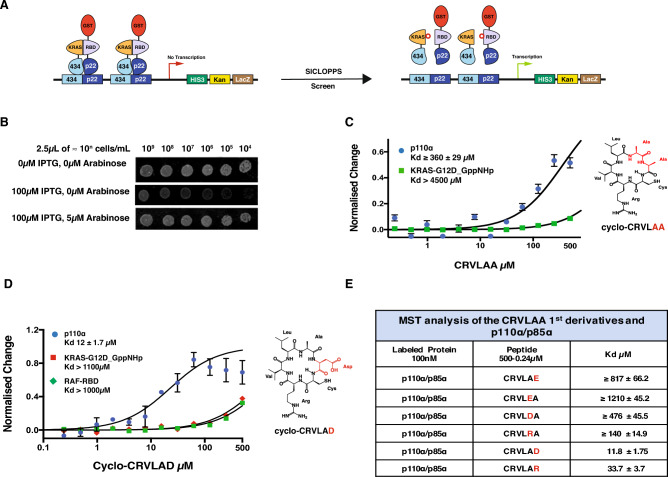
The RTHS and characterisation of cyclo-CRVLAA and its 1st derivatives. (**A**) A schematic presentation of the GST-RBDα/KRAS RTHS. The presence of GST at the N-terminal of the RBDα is to increase the domain’s solubility. The RTHS is controlled by the addition of IPTG which stops the growth of the bacterial cell on selective media upon the interaction between GST-RBDα and KRAS. Growth is recovered if a co-expressed cyclic peptide blocks the GST-RBDα/KRAS interaction by either binding to GST-RBDα or KRAS. (**B**) Bacterial drop spotting of the GST-RBDα/KRAS RTHS: bacteria grow normally in the absence of IPTG (GST-RBDα and KRAS are not expressed) upper lane; in the presence of 100 µM IPTG the RTHS shows lack of growth on higher dilutions, middle lane; in the presence of IPTG and arabinose (which controls expression of the cyclic peptides), where there is a peptide that blocks the protein interaction, the RTHS regains growth on selective media—as in the case with cyclo-CRVLAA bottom lane. (**C**) MST analysis of cyclo-CRVLAA with p110α (p110α/p85α) and KRAS. The cyclo-CRVLAA shows weak binding to p110α but no significant binding to KRAS. (**D**) The peptide derivative cyclo-CRVLAD interacts 30-fold more strongly with p110α than cyclo-CRVLAA but does not interact with KRAS or the CRAF-RBD. (**E**) Table displaying the MST analysis of all the cyclo-CRVLAA 1st derivatives with p110α (p110α/p85α).

The SICLOPPS technique as previously published is based on two steps: first, building a reverse two-hybrid system (RTHS) that expresses the target protein complex in bacteria as a screening platform and second, the production of a genetically encoded cyclic peptide library^25–29^. In designing the RTHS system, we chose to express the activated mutant KRAS-G12D (hereafter KRAS) together with the RBD of p110α (hereafter RBDα), as this is the domain of p110α that binds to KRAS. Also, the expression of the full length p110α needs to be in association with the regulatory subunit p85α in order to achieve a stable protein dimer, which makes it a large and complex protein to express in bacteria. In a successful RTHS system, the engineered bacterial cell line should show a reduction of growth on selective media upon induction of expression of the target protein complex with increasing isopropyl β-d-1-thiogalactopyranoside (IPTG)^27–29^. When designing the RTHS that expresses the RBDα/KRAS, we found that the reduced growth on selective media with IPTG was not strong enough to be used as a screening platform (Supplementary Fig. 1). We hypothesised that this could be due to a lack of solubility of RBDα, a previously described feature of the RBDs of p110 proteins^32^. Therefore, we designed another RTHS carrying a GST fusion tag at the N-terminus of the RBDα (GST-RBDα/KRAS RTHS). We found that the GST-RBDα/KRAS RTHS produced a stronger growth reduction on selective media with IPTG, making it a more suitable screening platform (Fig. 1A,B, Supplementary Fig. 1).

We used the GST-RBDα/KRAS RTHS to screen a CXXXXX (where X is any of the 20 canonical amino acids) SICLOPPS plasmid library of 3.2 million genetically encoded cyclic hexapeptides^25^. The cyclic peptides contain an invariant cysteine and five other amino acids that are each equally distributed between the twenty possibilities. Thus, each SICLOPPS plasmid contains a different sequence representing a unique peptide. We performed electroporation as described in detail in Tavassoli et al.^25^ and obtained a transformation efficiency of 10^10^, which suggests that we covered the library more than tenfold (Supplementary Fig. 2).

After several rounds of screening, we observed a regrowth of the GST-RBDα/KRAS RTHS (on selective media with IPTG) upon the arabinose induced expression of SICLOPPS plasmids encoding several cyclic peptides cyclo—(CRVLAA, CAWCGR, CLWYWW, CKLVVL, CVILVV, CRIVVI, CMSGGR, CWLLYL and CLVWWY) indicating that these peptides are able to block the RBDα/KRAS protein interaction. We selected cyclo-CRVLAA as the peptide of primary interest due to the fact that it appeared more than once as a positive hit in our screening—providing reassurance that it is a true positive and that we obtained good coverage of the SICLOPPS library (Fig. 1B, Supplementary Table 1). Moreover, cyclo-CRVLAA is the least hydrophobic of the primary hits, making it more straightforward to synthesise chemically and to solubilise, thereby enabling effective downstream analysis.

### Cyclo-CRVLAA and its derivatives interact specifically with p110α

To further understand how the cyclo-CRVLAA peptide blocks the RBDα/KRAS protein interaction, we synthesised this cyclic peptide for biophysical studies. Due to the poor solubility of the RBDα in vitro, we chose to use purified full-length p110α in complex with the regulatory subunit p85α expressed in baculovirus and KRAS expressed in bacteria for our biophysical analysis^6,32^. We chose Microscale Thermophoresis (MST) as a tool to detect the cyclo-CRVLAA interaction with either KRAS or p110α/p85α (hereafter p110α), for its sensitivity in detecting strong and weak interaction using small amounts of protein. The MST experiments showed that the cyclo-CRVLAA did not interact with KRAS but interacted weakly with p110α with an affinity around K_d_ ≥ 360 µM (Fig. 1C).

The technique of alanine scanning is often applied to amino acid-based inhibitors, such a peptides and DARPins (Designed Ankyrin Repeat Proteins), in order to detect the critical residues on the inhibitor that are involved in the interaction with the target protein^24,33^. Because the potential inhibitor cyclo-CRVLAA contains two alanine residues at positions 5 and 6, we sought to replace each of these with charged amino acids to increase the peptide solubility and to test if these changes could increase the peptide’s affinity for the p110α protein. Therefore, we synthesised 6 peptide derivatives of cyclo-CRVLAA (cyclo-CRVLAD, cyclo-CRVLDA, cyclo-CRVLAR, cyclo-CRVLRA, cyclo-CRVLEA, cyclo-CRVLEA) the physical properties of which are summarised in Supplementary Table 1. We applied the MST technique to test the peptide derivatives binding with p110α. Interestingly we found that cyclo-CRVLAD showed a 30-fold increase in affinity with p110α K_d_ 12 ± 1.7 µM (Fig. 1D,E). We also detected a tenfold increase in the interaction of cyclo-CRVLAR with p110α K_d_ 33.7 ± 3.7 µM (Fig. 1E, Supplementary Fig. 3A). These data indicate that position 6 in cyclo-CRVLAA favours a charged residue when alanine is present at position 5 of the peptide. The other peptide derivatives showed little or no improvement over cyclo-CRVLAA in their affinity for p110α (Fig. 1E, Supplementary Fig. 3B–E). It was of interest to note the difference between the binding affinities of cyclo-CRVLAD and CRVLAE, as both contain negatively charged amino acids at position 6. This could be due to the carboxylic acid side-chain of the aspartic acid (Asp) being involved in forming hydrogen bonds with p110α and extending this side-chain by one carbon in glutamic acid (Glu) could have destructive effect on the binding site. In addition, there may also be a relatively minor effect from the Glu residue on the backbone conformation of the cyclic peptide, which would in turn affect the binding of the rest of the interacting side chains of the peptide to the protein.

To test the specificity of the new peptide derivative cyclo-CRVLAD, we analysed if the peptide interacted with KRAS or CRAF-RBD using MST. We found that both KRAS and CRAF-RBD did not produce a positive signal of interaction with cyclo-CRVLAD, showing that the peptide is specific to p110α (Fig. 1D). It is also worth mentioning that all the peptide derivatives described above were more soluble than cyclo-CRVLAA.

**Figure 2:**
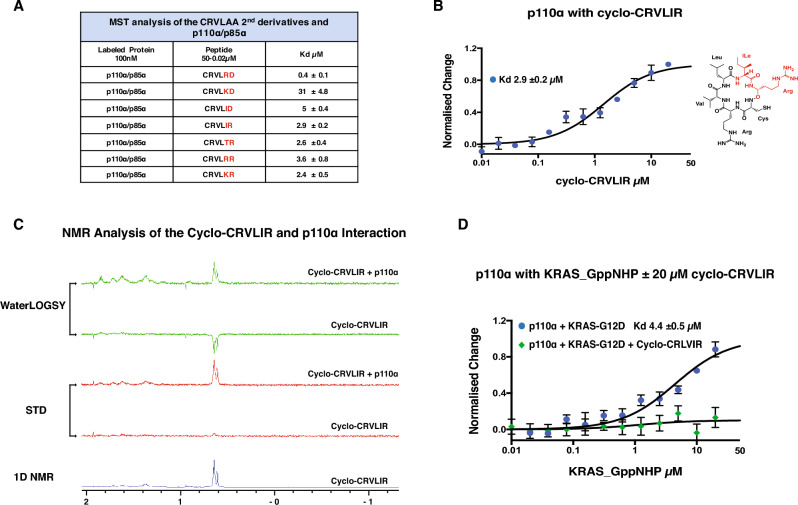
Characterisation of the 2nd cyclo-CRVLAA derivatives. (**A**) Table displaying the MST analysis of the 2nd cyclo-CRVLAA derivatives with p110α (p110α/p85α). (**B**) MST analysis of cyclo-CRLVIR with p110α (p110α/p85α). (**C**) NMR analysis of the interaction of cyclo-CRVLIR with 110α using WaterLOGSY and STD. (**D**) Competition assay using MST analysis of the p110α/KRAS interaction in the absence and presence of 20 µM cyclo-CRVLIR. p110α interacts with KRAS with an affinity of around K_d_ 4.4 µM and this interaction is blocked by the addition of 20 µM cyclo-CRVLIR.

Based on the above data, we synthesised a second round of peptide derivatives of cyclo-CRVLAA by modifying the first-round peptides cyclo-CRVLAD and cyclo-CRVLAR to carry neutral amino acids in position 5 of the peptide—to further enhance the peptide solubility. These new peptide derivatives are cyclo—(CRVLIR, CRVLID, CRVLRD, CRVLRR, CRVLKR, CRVLTR, CRVLKD) the physical properties of which are summarised in Supplementary Table 1. We analysed the binding affinities of the peptides with p110α and found a significant increase in the binding affinity with the exception of cyclo-CRVLKD (K_d_ 31. ± 4.8 µM) (Fig. 2A, Supplementary Fig. 4). Here we focus on derivative cyclo-CRVLIR, that interacts with p110α with a K_d_ of 2.9 ± 0.2 µM, due to its cellular activity that is presented later in this study (Fig. 2B). It is also worth pointing out that cyclo-CRVLIR containing Ile at position 5 could be less soluble than the previous derivative that contained an Ala in position 5 cyclo-CRVLAR. However, cyclo-CRVLIR was more soluble than the parent peptide cyclo-CRVLAA, also, the presence of Ile in position 5 increased the affinity cyclo-CRVLIR towards p110α protein.

### Cyclo-CRVLIR interacts with p110α and blocks the p110α/KRAS interaction in vitro

When studying PPI or protein/compound interactions, it is important to detect the interactions using complementary techniques that possess different characteristics. Here we chose Nuclear Magnetic Resonance (NMR) as an orthogonal technique to detect the cyclo-CRVLIR binding to p110α. We applied two commonly used NMR-based techniques that can detect protein/ compound interaction: STD (Saturation transfer difference), and Water-LOGSY (water-ligand observed through gradient spectroscopy)^34,35^. We analysed a solution of 1 mM of cyclo-CRVLIR peptide using STD and Water-LOGSY in the presence and absence of 8 µM p110α protein and observed a clear signal of interaction between cyclo-CRVLIR and p110α (Fig. 2C).

We next wanted to test whether the cyclo-CRVLIR peptide is capable of inhibiting the p110α/KRAS interaction. Using Isothermal titration calorimetry (ITC), it has been previously reported that the binding of p110α to KRAS occurred with a K_d_ of 3 µM^6^. We repeated the p110α/KRAS interaction using MST and obtained a very similar K_d_ of 4.4 ± 0.5 µM (Fig. 2D). Further, in the presence of 20 µM cyclo-CRVLIR we did not detect the p110α/KRAS interaction by this technique, which indicates that the peptide does indeed block the p110α/KRAS protein interaction (Fig. 2D).

### Cyclo-CRVLIR inhibits AKT phosphorylation in cancer cell lines

**Figure 3:**
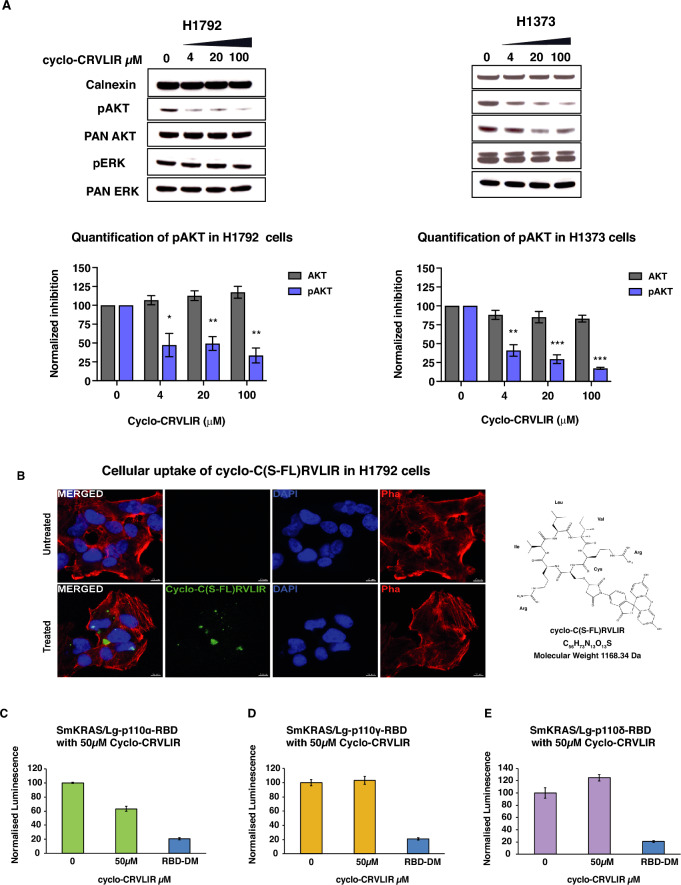
Analysis of the effect of cyclo-CRVLIR in cancer cell lines and NBBA. (**A**) H1792 and H1373 cells were treated with increasing concentrations of cyclo-CRVLIR (4, 20 and 100 µM) for 4 h. Cell lysates were probed with the indicated antibodies. Bottom graphs show expression of phospho-AKT (anti-pAKT-S473) and total AKT (normalised to calnexin expression). Mean ± SEM, N = 3, un-paired Student’s t-test treated vs untreated cells. Original blots with multiple exposure times are presented in Supplementary Fig. 6 with the main blot presented in Fig. 3A red box. (**B**) Cellular uptake of the fluorescein-conjugated cyclo-C(S-FL)RVLIR in H1792 cells. Representative images of H1792 cells, stained for DAPI (blue) and Phalloidin (red), after treatment with 100 μM of the peptide (green) for 24 h, on the right is the structure of the fluorescein-conjugated C(S-FL)RVLIR. (**C**–**E**) Testing the specificity of Cyclo-CRVLIR to RBDα using the NBBA. The three RAS binding domains of PI3K isoforms (Lg-RBDα, Lg-RBD δ and Lg-RBDγ) were transfected with Sm-KRAS in HEK293 cells, and cell lysates were treated with 50 µM cyclo-CRVLIR. Only Sm-KRAS/Lg-RBDα showed reduction in the interaction signal and not the other RBDs, demonstrating that cyclo-CRVLIR is an RBDα specific peptide. RBD-DM (a p110α-RBD with two mutations, T208D and K227A, that does not bind to RAS) was cloned and expressed in the Lg-BiT (Lg-RBD-DM). In the control experiments, Sm-KRAS-G12C was co-transfected with Lg-RBD-DM and the lysate was used as a negative control to indicate the true signal reduction upon the inhibition of the RAS/p110α interaction.

We tested whether the cyclo-CRVLIR peptide, as well as the other cyclo-CRVLAA 2nd derivatives (cyclo—CRVLID, CRVLRD, CRVLRR, CRVLKR, CRVLTR, CRVLKD), would be able to inhibit the p110α/KRAS interaction in cancer cell lines. We treated the human lung cancer cell line H1792, growing at steady-state in Roswell Park Memorial Institute (RPMI) 1640 media with 10% Foetal Calf Serum (FCS), with the seven cyclic peptides at three different concentrations (2, 10 and 50 µM) for 4 h. After treatment we measured the effect of the peptides on the phosphorylation levels of AKT (pAKT-Ser473), a key downstream effector of p110α activity. We found that cyclo-CRVLIR had a dose dependent effect on the levels of p-AKT, whereas the rest of the peptides showed almost no effect on p-AKT (Supplementary Fig. 5A,B). We confirmed the results obtained in H1792 cells using different doses of cyclo-CRVLIR and further validated the inhibition of pAKT in an alternative cell line, H1373 (Fig. 3A). In contrast, the phosphorylation levels of ERK (p-ERK), which is a downstream effector of the RAS/RAF/MEK/ERK pathway and is independent of p110α activity, were not affected. These data suggest that cyclo-CRVLIR action is specific to p110α and does not interfere with the RAS/RAF interaction or non-specifically impact on cellular function.

In order to demonstrate cellular uptake of the of cyclo-CRVLIR we synthesised a fluorescein-conjugated cyclo-C(S-FL)RVLIR peptide. Upon treatment of H1792 cells with this fluorescent peptide at 100 µM we readily observed the presence of cyclo-C(S-FL)RVLIR in the cytosol (Fig. 3B).

### Cyclo-CRVLIR is specific to p110α and not other PI3K isoforms

We next sought to determine whether cyclo-CRVLIR binds specifically to p110α or if it can also interact with other PI3K isoforms. We chose the NBBA (NanoBiT Biochemical Assay), which was previously developed by our laboratory, as a tool to detect if cyclo-CRVLIR could inhibit the interaction of RAS with p110α, p110 δ and p110γ^36^. The NBBA is biochemical adaptation of the NanoBiT cellular assay that is based on the split luciferase NanoLuc system that uses separate Large-BiT (Lg-BiT) and Small-BiT (Sm-BiT) components, each fused to a protein of interest. When the Lg-BiT and Sm-BiT proteins are brought into close proximity, upon the binding of their cognate target proteins, they produce a luminescence signal. We transfected HEK293 cells with plasmid constructs directing the expression of Sm-BiT fused to KRAS (Sm-KRAS) together with Lg-BiT fused to the RBD of either p110α, p110 δ or p110γ (Lg-p110α, Lg-p110 δ and Lg-p110γ). The p110β isoform was omitted from this analysis because it does not bind to RAS sub-family proteins^6,36^. Cells were harvested at 48 h post-transfection and the cell lysates tested for the interaction of Sm-KRAS with each of the PI3K binding isoforms in the presence or absence of 50 µM cyclo-CRVLIR. We found that cyclo-CRVLIR was able to inhibit the interaction of Sm-KRAS/LgRBDα but not Lg-RBD δ or Lg-RBDγ (Fig. 3C–E). These results show that cyclo-CRVLIR is specific for the RBD of p110α and not the other PI3K isoforms.

### Modelling of the cyclo-CRVLIR and molecular docking on the p110α-RBD

**Figure 4:**
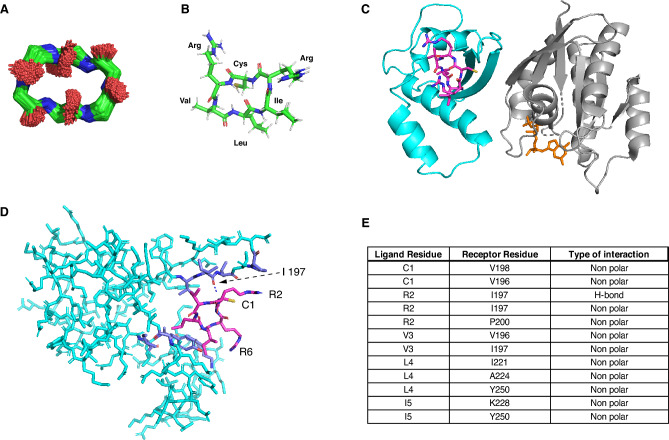
Modelling of the cyclo-CRVLIR and molecular docking on the p110α-RBD. (**A**) Illustration of the backbone conformations of cyclo-CRVLIR during a 10 ns Molecular Dynamic Simulation (MDS). (**B**) Proposed model of cyclo-CRVLIR structure based on the MDS studies. (**C**) A cartoon model based on previous published structure of GTP bound (orange) HRAS (grey) to p110γ (PDB: 1HE8). A previously published structure of p110α (cyan) (PDB: 4TV3) was aligned on top of the p110γ to represent a binding model of p110α with RAS protein^8,37^. The model shows the binding of cyclo-CRVLIR (magenta) to p110α at a site that is not involved in the binding with RAS—indicating allosteric inhibition of the p110α/RAS interaction. It is worth emphasising that the presence of RAS in this model is a representation to help indicate that the peptide binds to the RBD allosterically and not through he RBD/RAS interface, (**D**) Representation of cyclo-CRVLIR bound to p110α-RBD and possible formation of hydrogen bonds between R2 of cyclo-CRVLIR and I197 in p110α (black dashed lines). This also shows that R6 is not involved in the binding of the peptide to p110α, although it could be playing a major role in cellular penetration (**E**) A table showing each amino acid of cyclo-CRVLIR and its possible binding residues in the p110α pocket.

In order to gain a better understanding of cyclo-CRVLIR structure, we generated a model of the cyclic hexapeptide in silico by adjusting the torsion angles of a linear hexapeptide in order to satisfy the geometric constraints of a peptide bond between the N and C termini. The peptide was then optimised with energy minimisation using CHARMM force field. A 10 ns molecular dynamics simulation at 300 K in explicit water was performed to assess the stability of the peptide and determined that the backbone does not significantly change conformation over this simulation (Fig. 4A,B).

In order to further our theoretical understanding of how cyclo-CRVLIR could be binding to p110α and how this blocks its binding to KRAS, we applied molecular docking of the peptide on the previously published structure of p110α (PDB:4TV3) and aligned to the previously published HRAS/p110γ complex (PDB: 1HE8)^8,37^.

The docking of the cyclo-CRVLIR on the p110α was performed with the SwarmDock program in flexible mode, optimising the best binding pose concurrently with a linear combination of the five lowest frequency normal modes of the RBD. This permits the simulation of the flexibility of the domain according to Aramini et al^38^.

The complex presents cyclo-CRVLIR bound on top of the RBD between the α helices and the β sheets indicating that cyclo-CRVLIR binds to the RBD outside the binding interface with RAS. This suggests that cyclo-CRVLIR allosterically inhibits the p110α/KRAS protein–protein interaction (Fig. 4C). We observe that cyclo-CRVLIR forms a non-polar interaction with the RBD. There is a backbone-backbone hydrogen bond interaction between the R in position two of the cycle peptide (R2) and I197 of p110α, with the rest of the cyclic peptide residues forming hydrophobic interactions with the pocket in the RBD (Fig. 4D). From the model we can predict that the presence of both arginines play more of a role in the cyclic peptide solubility and cell penetration than potency towards the RBD. A summary of the cyclic peptide residue interactions with the RBD amino acids is listed in the table in Fig. 4E.

## Discussion

Inhibiting the function of oncogenic RAS proteins has historically been very challenging for a number of reasons. All RAS isoforms share identical N-terminal protein sequences, from amino acid 1–86, which contains the effector binding region with which RAS interacts with downstream effectors such as PI3K and RAF. This makes it very difficult to identify inhibitors that are isoform specific. In fact, most of the reported RAS inhibitors are selective for either all isoforms or for one specific type of oncogenic RAS mutation^14–19^. Pan RAS inhibitors carry the risk of causing unacceptable in vivo toxicity as they target all isoforms of both oncogenic and wild type RAS proteins. The only RAS inhibitors that have managed to reach the clinic are the covalent inhibitors that target the oncogenic RAS-G12C mutation^17–19^.

One possible alternative route to inhibiting oncogenic RAS activity is by inhibiting its interaction with downstream effector binders such as PI3K and RAF^6,10,39^. Previous reports have shown that blocking the p110α/RAS interaction had a strong inhibitory effect on the activity of oncogenic RAS in mice^20–22^. This made blocking the p110α/RAS–PPI a plausible drug discovery target. Proteins usually co-interact through a large surface which makes it difficult for a small molecule to inhibit successfully. Therefore, cyclic peptides present an attractive scaffold for inhibiting PPIs due to their size and geometry of their structure^31,40,41^. Indeed, there are successful examples of finding RAS cyclic peptide inhibitors such as cyclorasin 9A5 and KRpep-2d^42,43^. However, cyclorasin 9A5 is a Pan RAS inhibitor and KRpep-2d is specific only for KRAS-G12D. Therefore, discovering a cyclic peptide that binds to the RBDα and blocks the RAS/p110α interaction provides a wider range of usage as it can be employed against any oncogenic RAS mutant and not just one specific mutant. Given that blocking the p110α/RAS interaction in adult wild type mice showed no significant toxicity, such a reagent may also prove to be well-tolerated in an in vivo setting^20,21^.

In our study we chose the SICLOPPS technique for its robustness in producing and screening cyclic peptides that have been shown to be effective in inhibiting PPIs^23,26,28,29,44^. We applied a SICLOPPS library of around 3.2 million peptides to screen for cyclic peptide inhibitors that block the p110α/RAS interaction and isolated several cyclic peptides. However, cyclo-CRVLAA was the most soluble of these and appeared in several bacterial colonies as a positive hit. In addition, cyclo-CRVLAA interacted with p110α rather than KRAS, which was one of the primary goals of the screen. Moreover, after two steps of alanine substitution, we discovered that a modified version, cyclo-CRVLIR interacts specifically with p110α with an almost 120-fold stronger affinity than cyclo-CRVLAA. More interestingly, cyclo-CRVLIR also inhibits the p110α/RAS interaction and reduces the levels of pAKT in H1792 and H1373 cancer cell lines.

An interesting question is why cyclo-CRVLIR was not identified as a hit from the primary screening process, and there are a number of viable explanations. In the SICLOPPS system, it is possible that some of the expressed cyclic peptides may be toxic to the bacterial cells and affect their growth. Alternatively, it could be that certain peptide sequences do not accurately splice to form the cyclic peptide^25,26,31^. Thus, the actual diversity of expressed cyclic peptides is hard to predict as it is not possible to know in advance which peptides may prove toxic or not accurately spliced in addition to those containing a stop codon within the CXXXXX core sequence. Nevertheless, the actual diversity of the library will clearly be less than the mathematically calculated 3.2 million.

The affinity of interaction of cyclo-CRVLIR with p110α (K_d_ 2.9 µM) may be insufficiently strong to be useful as an immediate p110α inhibitor and further optimisation would be required for purposes beyond its utility as a tool compound. However, cyclo-CRVLIR represents a first stage towards the development of an inhibitor of the p110α–RBD interaction, with strong potential to be used as a starting point for development of a more potent p110α binder. Cyclo-CRVLIR is a small 6 residue cyclic peptide with a molecular weight of around 0.76 kDa, similar to small molecule inhibitors, and the ability to penetrate cellular membranes, as presented in our results. This could be due to a combination of the fact that it contains two arginine residues, which may help promote cellular penetration, as well as its affinity towards the p110α protein. In addition, cyclo-CRVLIR is significantly more soluble than our original cyclo-CRVLAA peptide and can be dissolved in aqueous buffers, reducing possible vehicle effects when used at higher concentrations. We also show through our modelling that cyclo-CRVLIR binds to p110α-RBD to allosterically inhibit its interaction with KRAS. Allosteric inhibition of p110α activity has been previously shown for the BYL-719 small molecule inhibitor which stabilises the p110α interaction with its regulatory subunit p85α, blocking binding of p110α to the membrane and interfering with access of ATP and phosphoinositides^45^. Further efforts to build on our findings using cyclo-CRVLIR are ongoing and include extended analysis of the proposed binding model of cyclo-CRVLIR with the p110α-RBD and the substitution of natural residues with non-natural amino acids. This could provide cyclo-CRVLIR with stronger potency towards p110α and greater stability in a cellular environment. Another interesting characteristic of cyclo-CRVLIR is selectivity towards p110α above the two other isoforms p110 δ and p110γ, which is not surprising giving that sequence similarity among the 3 isoforms is very low, specifically around the RBD. In summary, we report cyclo-CRVLIR as the first specific inhibitor of the interaction of RAS with p110α through binding to p110α-RBD. This has significant potential to be developed into a future p110α targeted drug for possible use in RAS driven cancers.

## Methods

### SICLOPPS: producing the RAS/p110α-RBD and KRAS-G12D

Due to the lack of solubility of the p110α-RBD we created a fusion of the RBD (amino acids 133 – 314) with GST (Glutathione S-transferase) by PCR amplification, to produce a recombinant protein of GST-RBD (hereafter RBD). Both KRAS (amino acids 1–168—lacking the Hyper Variable Region) and the RBD were cloned into the pTHCP14 vector that contains two bacteriophage repressors P22 and 434, producing a cassette that consists of two fusion proteins—KRAS with 434 and RBD with P22. This cassette was then sub-cloned into the CRIM integration plasmid pAH68—for integration into E. coli strain SNS126 that contains the chimeric P22 and 434 operators. When expressed, the interaction of KRAS with the RBD brings together the P22 and the 434 repressors to form a dimer that will interact with the chimeric P22 and 434 operators—resulting in no growth on selective media.

The SICLOPPS library was generated by PCR amplification using degenerate primers encoding cyclo-CXXXXX hexapeptides (where X is any amino acid), producing around 3.2 million compounds. The SICLOPPS RTHS formation, cyclic peptide production and library screening were followed as described^24,25,46^.

### Expression of PI3-kinase

Sf21 cell cultures at 1.5 × 10^6^ cells/ml were infected with high titre baculoviruses encoding a GST fused p110α subunit and full-length p85α at an MOI of 1. These cultures were allowed to grow for 72 h at 27 °C with constant rotation at 120 rpm. Cells were then harvested at stored-80 °C until required.

### Purification of PI3-kinase

Sf21 cell pellets from the Sf21cultures were thawed on ice and resuspended in 30 ml lysis buffer^6^ and cells were lysed by sonication. The resulting suspension was then centrifuged at 30,000 rpm to pellet insoluble material and the soluble fraction was incubated with 1 ml glutathione resin for 2 h at 4 °C with constant rotation. The resin was then washed extensively with 50 mM HEPES (pH 8.0), 200 mM NaCl, 1 mM DTT. To elute the kinase, the resin was resuspended in 5 ml of the same wash buffer supplemented with 3C protease and cleavage was allowed to occur overnight at 4 °C. Cleaved protein was separated from the resin by centrifugation (13,000 rpm for 10–15 min) and concentrated to 0.5 ml in a Vivaspin concentrator (MWCO 30,000 Da). This preparation was applied to a S200 10/300 size exclusion column equilibrated in cleavage buffer supplemented with 5% glycerol. Purified protein eluted as a single peak and relevant samples were analysed by SDS-PAGE. Fractions containing PI3-Kinase were pooled, quantified by UV spectroscopy and snap frozen at a concentration of approximately 3 µM.

### Peptide synthesis

Peptides were synthesised on an Activotec P11 peptide synthesiser (Activotec, Cambridge, UK) on pre-loaded LL Wang resin, using N(a)-Fmoc amino acids including Fmoc-Cys (StBu)-OH, with HATU as the coupling reagent. Following amino acid chain assembly, peptides were cleaved from the resin by addition of 10 ml cleavage cocktail (92.5% TFA, 2.5% H_2_O, 2.5% EDT, 2.5% TIS) for 2 h. Following resin removal, peptide precipitation and extensive washing with ether, the peptides were freeze dried. The crude linear peptides were cyclised with PyAOP (5 eq.) and DIPEA (10 eq.) in DMF (1 mg/mL). The reaction mixtures were stirred for 24 h, concentrated and purified on a C8 reverse phase HPLC column (Agilent PrepHT Zorbax 300SB-C8, 21.2 × 250 mm, 7 m) using a linear solvent gradient of 0–55% MeCN (0.08% TFA) in H_2_O (0.08% TFA) over 60 min at a flow rate of 8 ml/min.

The StBu protecting group was removed from Cys in the purified peptides by treatment with DTT (10 eq.) in DMF:0.1 M (NH_4_)_2_CO_3_ (1:1). The reaction was stirred for 2 h at RT under argon. Following a second purification as described, peptides were analysed by LC–MS on an Agilent 1100 LC-MSD and all MWs agreed with calculated.

### Synthesis of the fluorescein-conjugated cyclo-C(S-FL)RVLIR

Synthesis was as for the other cyclo-peptides above. After synthesis and removal of the StBu group, the cyclo 6.6 reaction mixture was purified on a C8 reverse phase HPLC column (Agilent PrepHT Zorbax 300SB-C8, 21.2 × 250 mm, 7 m) using a linear solvent gradient of 5–45% MeCN (0.08% TFA) in H2O (0.08% TFA) over 40 min at a flow rate of 8 mL/min. The purified peptide was analysed by LC–MS on an Agilent 1100 LC-MSD. The calculated MW of the peptide was in agreement with the mass found. The purified peptide was then fluoresceinated using fluorescein-5-maleimide. A freshly prepared sodium phosphate buffer was made using 28.3 mg (0.02 M) of sodium phosphate and 58.4 mg (0.15 M) NaCl in 10 ml water, pH 6.5–7.5. 2 mg of cyclo 6.6 peptide (MW 740.98, 1 eq) was dissolved in 1 ml of fresh sodium phosphate buffer to which 5 µl of ethanedithiol was added. 10 mg of fluorescein-5-maleimide (MW 427.4, 8.6 eq) were dissolved in 1 ml DMF and added to the peptide reaction mixture, stirred for 2 h at RT then analysed by LC–MS. The reaction products were purified on a C8 reverse phase HPLC column (Agilent PrepHT Zorbax 300SB-C8, 21.2 × 250 mm, 7 min) using a linear solvent gradient of 15–75% MeCN (0.08% TFA) in H_2_O (0.08% TFA) over 40 min at a flow rate of 8 mL/min. The purified peptide was analysed by LC–MS on an Agilent 1100 LC-MSD. The calculated MW of the peptide was in agreement with the mass found.

### Microscale thermophoresis (MST)

Measurements were performed using a NanoTemper Monolith™ NT.115 instrument (NanoTemper Technologies GmbH, München, Germany). Protein samples were labelled with the amine reactive dye NT-647 (or NT-495) using the Monolith™ NT.115 Protein Labeling Kit RED-NHS (or Monolith™ NT.115 Protein Labeling Kit BLUE-NHS). Labelling levels (generally in the range 0.3–0.4 dye molecules per protein molecule) were determined using calculated extinction coefficients for the protein and ε647 = 250,000 M^−1^ cm^−1^ (or ε495 = 70,000 M^−1^ cm^−1^) for the dye concentration. In a typical experiment 20 ml aliquots of a 100 nM stock solution of labelled protein were mixed with 20 ml aliquots of a serial dilution of binding partner. These solutions were then loaded into standard treated capillaries and MST measurements were performed at 25 °C using 20–40% LED power and 40–60% IR-Laser power. The laser Laser-On time was 30 s and Laser-Off time 5 s. All measurements were performed at least five times.

### NMR

NMR experiments were performed at 20 °C using a Bruker Avance IIIHD spectrometer operating at 700 MHz. STD experiments employed off-resonance saturation (2 s) at − 40 ppm, and on-resonance at − 1 ppm. Waterlogsy experiments used a mixing time of 1.5 s.

### Cancer cell line treatment

Human Non-Small Cell Lung Cancer cell lines, H1792 and H1373 were seeded in 6 well plates at a density of 3.5 × 10^5^ cells per well and incubated overnight in 2 mL RPMI 1640 medium containing 10% Foetal Calf Serum (FCS) to induce phospho-ATK. The next day, 1 ml of medium was removed and the cells were treated with different peptide concentrations for 4 h. All peptides were dissolved in 25 mM Tris pH8, 100 mM NaCl and 1 mM TCEP. Cells were then harvested and lysed for SDS-PAGE and western blot analysis. Anti-calnexin antibody was obtained from Abcam. Antibodies directed against phospho-T202/Y204-ERK, ERK, phospho-S473-AKT and AKT were obtained from Cell Signaling. After western blotting, calnexin, AKT and phospho-AKT signals were quantified using ImageJ and statistical tests were done using Prism software.

### Immunofluorescence

H1792 cells were plated in 6-well plates containing 35 mm cover slips (VWR INTERNATIONAL-No. 631) at a density of 3.5 × 10^5^ cells per well. The next day cells were treated with 100 μM fluorescein-conjugated cyclo-RVLIP (cyclo-C(S-FL)RVLIP). After 24 h incubation at 37 °C with cyclo-C(S-FL)RVLIP, cells were fixed with Methanol-Free 4% Formaldehyde (Cell Signalling-47746). Cover slips were washed twice with DPBS before incubation with blocking solution (5% FCS, 1% BSA, 0.3% Triton-X, DPBS) and then stained with 1:2000 Alexa Fluor™ 594 Phalloidin (Invitrogen-A1238). After mounting slides with ProLong™ Gold Antifade Mountant (Thermo Fisher-P36935) with DAPI, cells were visualised at × 63 magnification using a Zeiss Upright710 confocal microscope. Images were generated using Imaris software.

### Nanobit biochemical assay (NBBA)

The production of the Sm-KRAS, and the Lg-RBD- of p110α, β, δ, γ constructs and lysate are described in detail in a previous report^36^. The cell lysate was treated with 50 µM cyclo-CRVLIR for 20–30 min before detection by the addition of the Nano-Glo.

### Molecular modelling of the hexapeptide CRVLIR

The cyclic peptide was first generated ab initio as a linear peptide using CHARMM internal coordinate parameters^47^. The in silico cyclisation was performed by adjusting the φ and ψ torsion angles of the residues in order to satisfy the geometric properties of a peptide bond between the N-terminal Cysteine and the C-terminal Arginine of the linear peptide. The adjustment was performed using a Particle Swarm Optimisation^52^, with pairs of φ and ψ angles emitted with a probability distribution corresponding to the distribution in the Ramachandran plot for each respective residue. The energy of the cyclic hexapeptide obtained was then minimised using GROMACS with OPLS/AA force field^48^. After minimisation, a short molecular dynamics simulation (10 ns) after NVT and NPT equilibration was run to obtain an energetically stable structure. The MD simulation was also performed with GROMACS.

### Molecular Docking of the hexapeptide CRVLIR on the RBD of p110α

The modelled hexapeptide was docked using the SwarmDock program using the DCOMPLEX energy potential, with normal modes linear combination to simulate the flexibility of the structure^49–51^. The SwarmDock protocol was run 200 times with different initial conditions and the solution selected corresponded to the largest cluster of docked poses with the lowest energy score. The model of p110α-RBD was then docked by homology (superimposition) to Ras, using Protein Data Bank (PDB), PDB: 1HE8 (HRAS-G12V/p110γ) as template. The RBD domain was taken from PDB:4TV3 of the p110α structure from the Protein Data Bank (PDB).

## Supplementary Information

Supplementary Information 1.

Supplementary Information 2.

Supplementary Information 3.

Supplementary Information 4.

Supplementary Information 5.

Supplementary Information 6.

Supplementary Information 7.

Supplementary Information 8.

Supplementary Information 9.

## Data Availability

The data supporting the findings of this study are available within the paper and its supporting information files. Raw data underpinning this study are openly available from the Francis Crick Institute in a Figshare repository (https://crick.figshare.com/).
