# Author Correction: Characterisation of a cyclic peptide that binds to the RAS binding domain of phosphoinositide 3-kinase p110α

**DOI:** 10.1038/s41598-025-91151-4

**Published:** 2025-03-13

**Authors:** Mohamed Ismail, Stephen R. Martin, Roger George, Francesca Houghton, Geoff Kelly, Raphaël A. G. Chaleil, Panayiotis Anastasiou, Xinyue Wang, Nicola O’Reilly, Stefania Federico, Dhira Joshi, Hemavathi Nagaraj, Rachel Cooley, Ning Sze Hui, Miriam Molina‑Arcas, David C. Hancock, Ali Tavassoli, Julian Downward

**Affiliations:** 1 Oncogene Biology Laboratory, Francis Crick Institute, 1 Midland Road, London, NW1 1AT UK; 2 Structural Biology, Science Technology Platforms, Francis Crick Institute, 1 Midland Road, London, NW1 1AT UK; 3 Peptide Chemistry, Science Technology Platforms, Francis Crick Institute, 1 Midland Road, London, NW1 1AT UK; 4 Biomolecular Modelling Lab, Francis Crick Institute, 1 Midland Road, London, NW1 1AT UK; 5 School of Chemistry, University of Southampton, Southampton, SO17 1BJ UK

Correction to: *Scientific reports* 10.1038/s41598-023-28756-0, published 02 February 2023

The original version of this Article contained an error.

As a result of an error during assembly of Fig. 3, the blot representing pan-AKT for H1373 (A) was duplicated from pan-ERK. The original Fig. 3 and accompanying legend appear below.

**Fig. 3:**
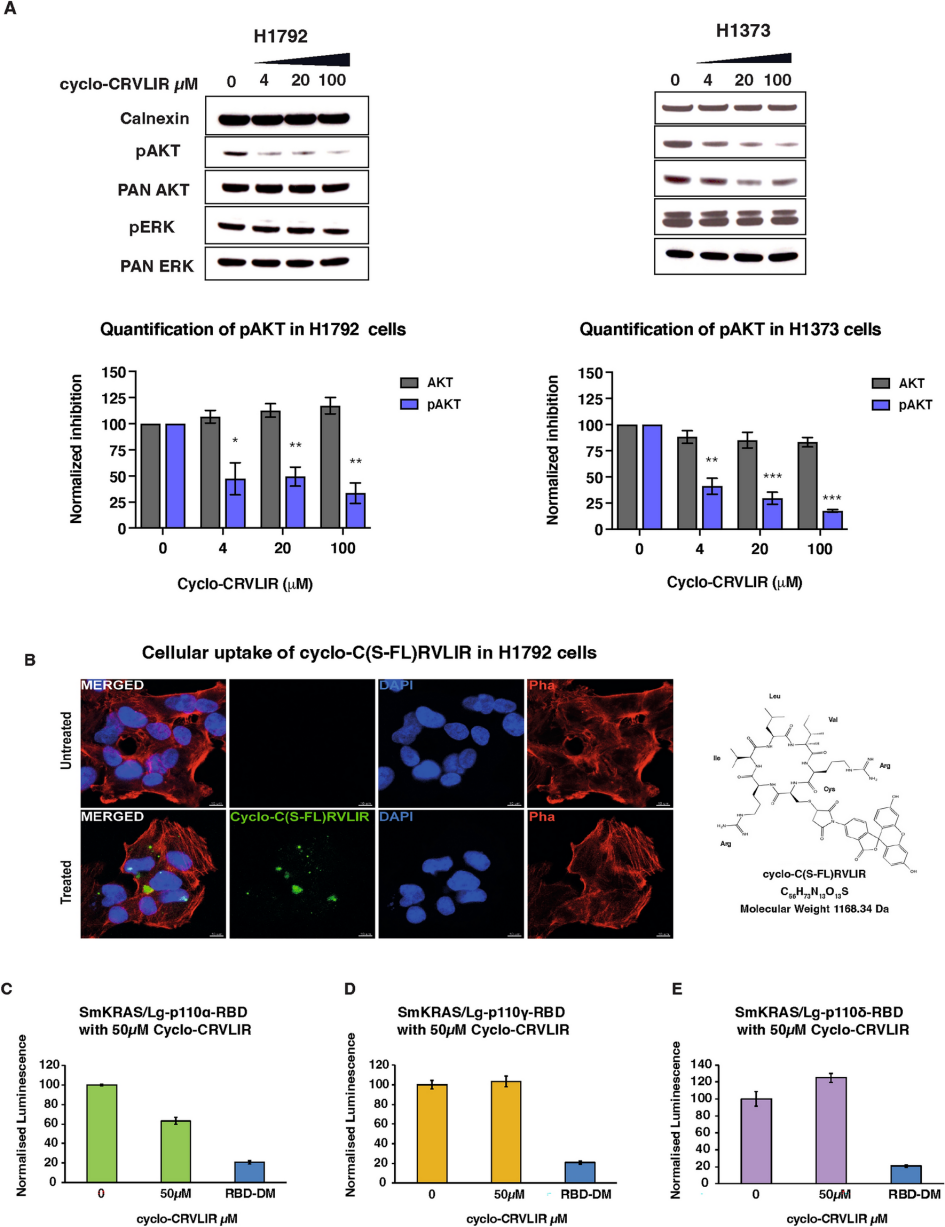
Analysis of the effect of cyclo-CRVLIR in cancer cell lines and NBBA. (**A**) H1792 and H1373 cells were treated with increasing concentrations of cyclo-CRVLIR (4, 20 and 100 µM) for 4 h. Cell lysates were probed with the indicated antibodies. Bottom graphs show expression of phospho-AKT (anti-pAKT-S473) and total AKT (normalised to calnexin expression). Mean ± SEM, N = 3, un-paired Student’s t-test treated vs untreated cells. Original blots with multiple exposure times are presented in Supplementary Fig. 6 with the main blot presented in Fig. 3A red box. (**B**) Cellular uptake of the fluorescein-conjugated cyclo-C(S-FL)RVLIR in H1792 cells. Representative images of H1792 cells, stained for DAPI (blue) and Phalloidin (red), after treatment with 100 μM of the peptide (green) for 24 h, on the right is the structure of the fluorescein-conjugated C(S-FL)RVLIR. (**C**–**E**) Testing the specificity of Cyclo-CRVLIR to RBDα using the NBBA. The three RAS binding domains of PI3K isoforms (Lg-RBDα, Lg-RBD δ and Lg-RBDγ) were transfected with Sm-KRAS in HEK293 cells, and cell lysates were treated with 50 µM cyclo-CRVLIR. Only Sm-KRAS/Lg-RBDα showed reduction in the interaction signal and not the other RBDs, demonstrating that cyclo-CRVLIR is an RBDα specific peptide. RBD-DM (a p110α-RBD with two mutations, T208D and K227A, that does not bind to RAS) was cloned and expressed in the Lg-BiT (Lg-RBD-DM). In the control experiments, Sm-KRAS-G12C was co-transfected with Lg-RBD-DM and the lysate was used as a negative control to indicate the true signal reduction upon the inhibition of the RAS/p110α interaction.

The original Article has been corrected.

