# Supplementary material for: Characterisation of a cyclic peptide that binds to the RAS binding domain of phosphoinositide 3-kinase p110α
Source: Sci Rep. 2023 Feb 2;13:1889. doi: 10.1038/s41598-023-28756-0 (PMC9894841; doi:10.1038/s41598-023-28756-0)
Supplement: Supplementary file 1 — Supplementary Information 1. [file 41598_2023_28756_MOESM1_ESM.docx]

**Supplementary Information**

**Characterisation of a cyclic peptide that binds to the RAS binding domain of phosphoinositide 3-kinase p110α**

Mohamed Ismail1, Stephen R. Martin2, Roger George2, Francesca Houghton1, Geoff Kelly2, Raphaël AG Chaleil ^4^, Panayiotis Anastasiou^1^, Xinyue Wang1, Nicola O’Reilly3, Stefania Federico3, Dhira Joshi^3^, Hemavathi Nagaraj^3^, Rachel Cooley1, Ning Sze Hui1, Miriam Molina-Arcas1, David C. Hancock1, Ali Tavassoli5 and Julian Downward1,6

**Supplementary Figure Legends**

**Supplementary Figure 1.** ONPG assay for the RBDα/KRAS and GST-RBDα/KRAS RTHS and the SNS126 cell line containing the repressor domains alone as control. The growth of the RTHS is reduced with the increasing concentrations of ONPG. The GST-RBDα/KRAS RTHS shows stronger growth suppression than RBDα/KRAS that lacks GST, which makes the GST-RBDα/KRAS RTHS more suitable as a screening platform.

**Supplementary Figure 2**. Images of petri dishes showing the bacterial growth on agar representing transformation efficiency of the SICLOPPS library through a 10 fold dilution series.

**Supplementary Figure 3.** (A-E) MST analysis of cyclo-CRVLAA 1^st^ derivatives with p110α, showing the variation of K_d_ obtained from each amino acid change to the alanine in position 5 or 6 of the cyclic peptide.

**Supplementary Figure 4.** (A-G) MST analysis of cyclo-CRVLAA 2^st^ derivatives with p110α protein. These peptides included a second amino acid change to the alanine in position 5 or 6 of the cyclic peptide. All peptides showed strong increase in the binding affinity for p110α protein.

**Supplementary Figure 5.** (A) H1792 cells were treated with three concentrations of cyclo-CRVLIR (2, 10 and 50µM) for 4 hours. Cell lysates were probed with the indicated antibodies. Graph shows expression of phospho-AKT and total AKT (normalised to calnexin expression). Mean+SEM, N=3, un-paired Student’s t-test treated versus untreated cells. Multiple time exposure of the original blots are presented in Supplementary Figure 7 with the blot presented in Supplementary Figure 5A in a red box. (B) H1792 cells were treated with different cyclo-CRVLAA 2^nd^ derivatives for 4 hours. Cell lysates were probed with the indicated antibodies – all peptides showed no significant effect on phospho-AKT levels with the exception of cyclo-CRVLTR that showed a mild effect. Original blots are presented in red boxes in Supplementary Figure 8.

**Supplementary Figure 6.** (A and B) Original western blot membranes presented in Figure 3 (in red box) with other multiple time exposures, representing the dose response of cyclo-CRVLIR (4, 20 and 100 µM) in H1792 and H1373 cells. (C and D) represents the original uncropped membranes of the same experiments.

**Supplementary Figure 7.** (A) Original western blot membranes presented in Supplementary Figure 5A in red box, with another time exposure, representing the dose response of cyclo-CRVLIR (2, 10 and 50 µM) in H1792 cells. (B) represents the original uncropped membranes of the same experiment.

**Supplementary Figure 8.** (A) Original western blot membranes presented in Supplementary Figure 5B, representing the dose response of several derivatives of cyclo-CRVLAA (2, 10 and 50 µM) in H1792 cells. (B) represents the original uncropped membranes of the same experiment.

**Supplementary table 1**

Physical properties of the synthesised cyclic peptides

| **Peptide** | **Sequence** | **Act Mwt** | **Calc MWT** | **Gravy** | **pI** |
| --- | --- | --- | --- | --- | --- |
| cyclo-CAWCGR | Cyclo-CAWCGR | 675.98 | 676.27 | 0.167 | 8.07 |
| cyclo-CLWYWW | Cyclo-CLWYWW | 936.99 | 937.41 | 0.383 | 5.52 |
| cyclo-CRLVLAA | Cyclo-CRLVLAA | 726.10 | 726.43 | 1.914 | 8.25 |
| cyclo-CKLVVL | Cyclo-CKLVVL | 655 | 655.42 | 2.433 | 8.22 |
| cyclo-CVILVV | Cyclo--CVILVV | 626.15 | 626.39 | 3.900 | 5.52 |
| cyclo-CRIVVI | Cyclo-CRIVVI | 683.12 | 683.43 | 2.567 | 8.25 |
| cyclo-CMSGGR | Cyclo-CMSGGR | 590.86 | 591.24 | -0.283 | 8.25 |
| cyclo-CWLLYL | Cyclo-CWLLYL | 790.93 | 791.41 | 1.95 | 5.52 |
| cyclo-CLVWWY | Cyclo-CLVWWY | 850.08 | 850.39 | 1.233 | 5.52 |
| cyclo-CRVLAA | Cyclo-CRVLAA | 612.96 | 613.35 | 1.600 | 8.25 |
| Cyclo 5-1 | Cyclo-CRVLAE | 671.05 | 671.35 | 0.717 | 5.99 |
| Cyclo 5-2 | Cyclo-CRVLEA | 671.03 | 672.38 | 0.717 | 5.99 |
| Cyclo 5-3 | Cyclo-CRVLDA | 657.01 | 657.34 | 0.717 | 5.83 |
| Cyclo 5-4 | Cyclo-CRVLAD | 657 | 657.34 | 0.717 | 5.83 |
| Cyclo 5-5 | Cyclo-CRVLRA | 698.21 | 698.41 | 0.55 | 10.35 |
| Cyclo 5-6 | Cyclo-CRVLAR | 698.23 | 698.41 | 0.55 | 10.35 |
| Cyclo 6.1 | Cyclo-CRVLRD | 742.16 | 742.91 | -0.333 | 8.25 |
| Cyclo 6.2 | Cyclo-CRVLKD | 714.20 | 714.90 | -0.233 | 8.22 |
| Cyclo 6.3 | Cyclo-CRVLID | 699 | 699.88 | 1.167 | 5.83 |
| Cyclo 6.4 | Cyclo-CRVLRR | 783.3 | 784.01 | -0.500 | 11.7 |
| Cyclo 6.5 | Cyclo-CRVLKR | 755.2 | 756.00 | -0.400 | 10.86 |
| Cyclo 6.6 | Cyclo-CRVLIR | 739.98 | 740.98 | 1.000 | 10.35 |
| Cyclo 6.7 | Cyclo-CRVLTD | 667 | 687.83 | 0.300 | 5.83 |
| Cyclo 6.8 | Cyclo-CRVLTR | 728.15 | 728.93 | 0.133 | 10.35 |
| Cyclo 6.6 | cyclo-CRVLIR | 740.2 | 740.98 | 1.000 | 10.35 |
| Fl-Cyclo 6.6 | cyclo-C(S-FL)RVLIR | 1167.81 | 1168.38 |  |  |
| Cyclo 6.6 | cyclo-CRVLIR | 740.62 | 740.98 | 1.000 | 10.35 |
| Cyclo 6.6 -Alexa647 | (cycloC(S-Alexa647)RVLIR) | 861.2 m/2 ion | 1720.84 |  |  |
| Cyclo 6.6 | cyclo-CRVLIR | 739.85 | 740.98 | 1.000 | 10.35 |
| Cyclo 6.6 | cyclo-CRVLIR | 740.04 | 740.98 | 1.000 | 10.35 |
| Cyclo 6.6 | cyclo-CRVLIR | 740.26 | 740.98 | 1.000 | 10.35 |

The physical properties of the cyclo-CRVLAA and its synthesized derivatives, describing the peptides sequences, MW (Molecular Weight), GRAVY (Grand Average of Hydropathicity Index) and PI (Isoelectric point).
