# Supplementary figures and images for: Characterisation of a cyclic peptide that binds to the RAS binding domain of phosphoinositide 3-kinase p110α
Source: Sci Rep. 2023 Feb 2;13:1889. doi: 10.1038/s41598-023-28756-0 (PMC9894841; doi:10.1038/s41598-023-28756-0)

Supplementary Figure 1

ONPG Assay

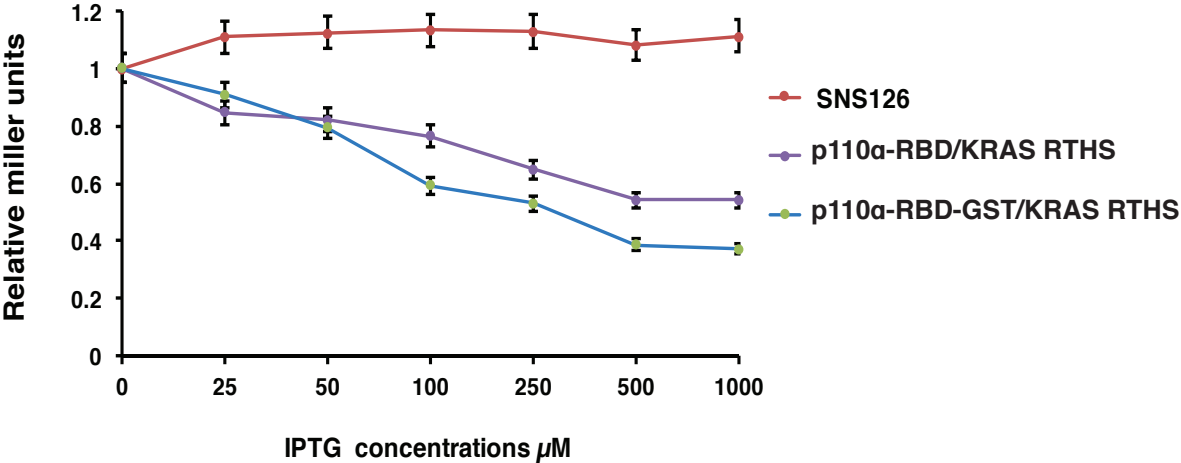

Supplement: Supplementary file 2 — Supplementary Information 2. [file 41598_2023_28756_MOESM2_ESM.pdf]

Supplementary Figure 2

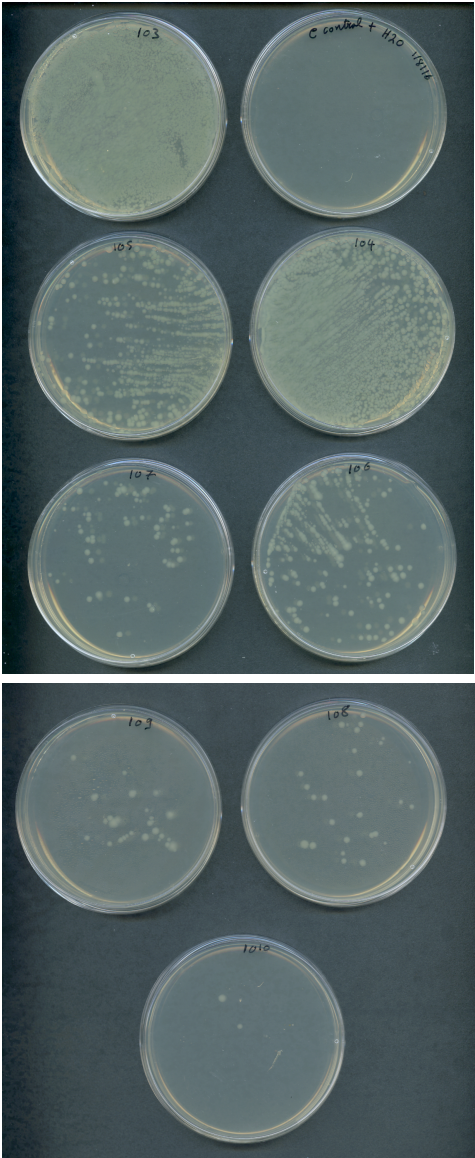

Supplement: Supplementary file 3 — Supplementary Information 3. [file 41598_2023_28756_MOESM3_ESM.pdf]

Supplementary Figure 3

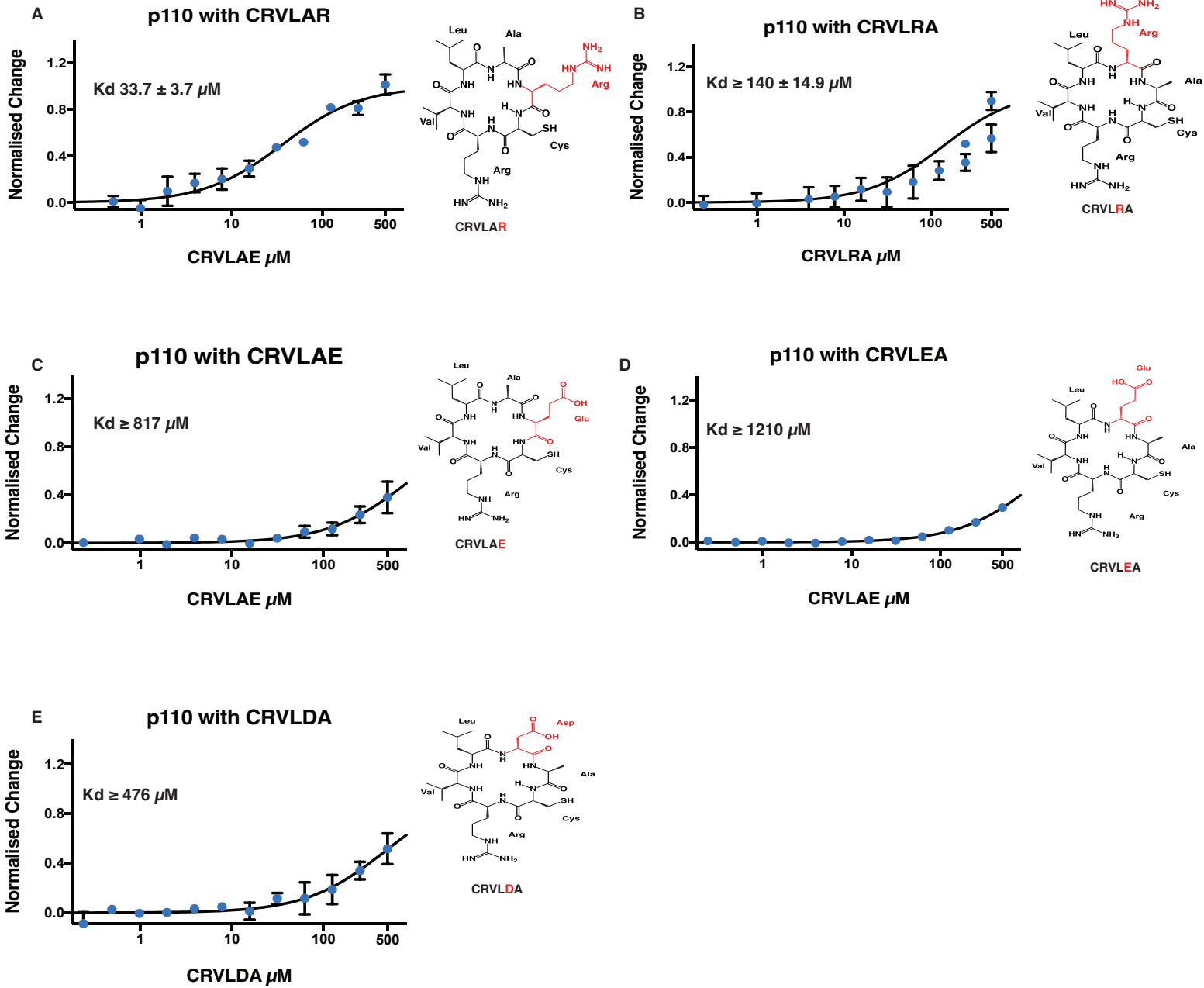

Supplement: Supplementary file 4 — Supplementary Information 4. [file 41598_2023_28756_MOESM4_ESM.pdf]

Supplementary Figure 4

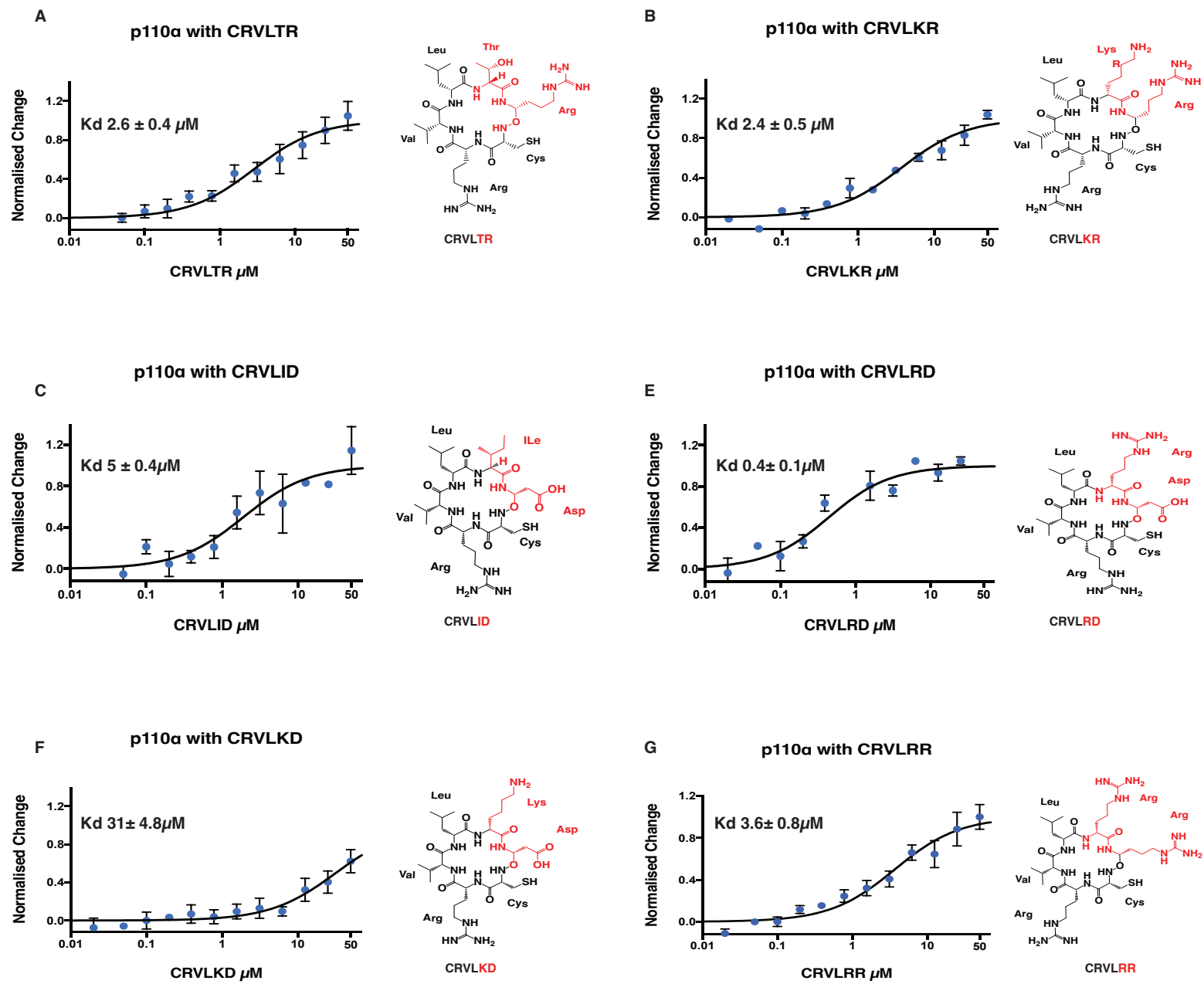

Supplement: Supplementary file 5 — Supplementary Information 5. [file 41598_2023_28756_MOESM5_ESM.pdf]

Supplementary Figure 5

A

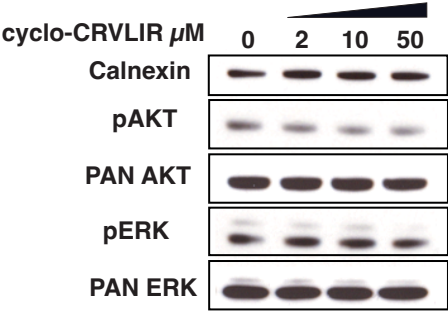

Quantification of pAKT in H1792 cells

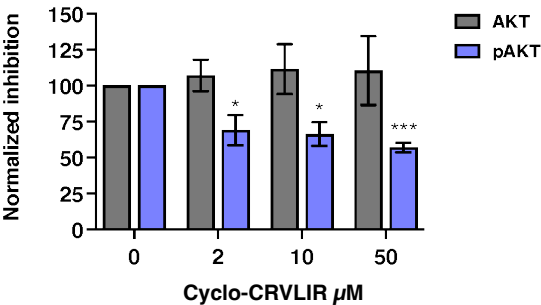

B

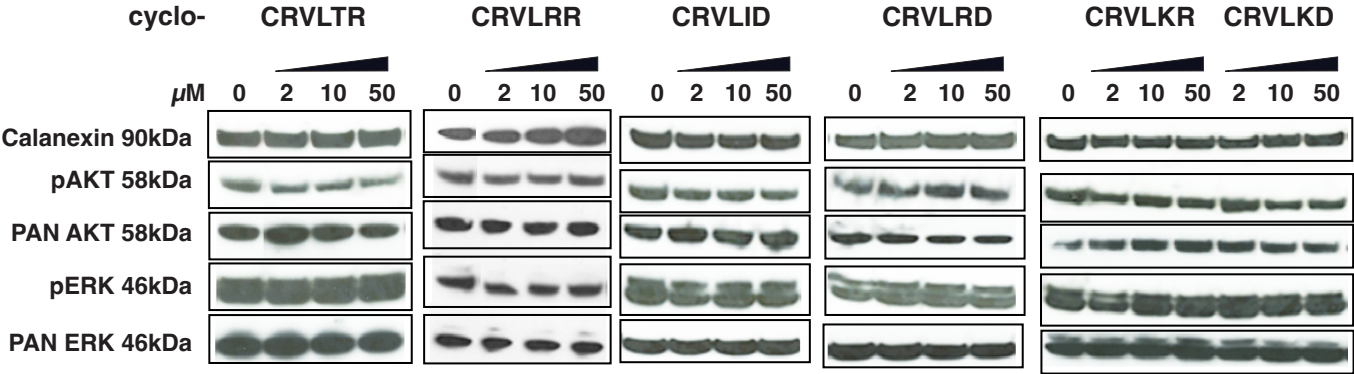

Supplement: Supplementary file 6 — Supplementary Information 6. [file 41598_2023_28756_MOESM6_ESM.pdf]

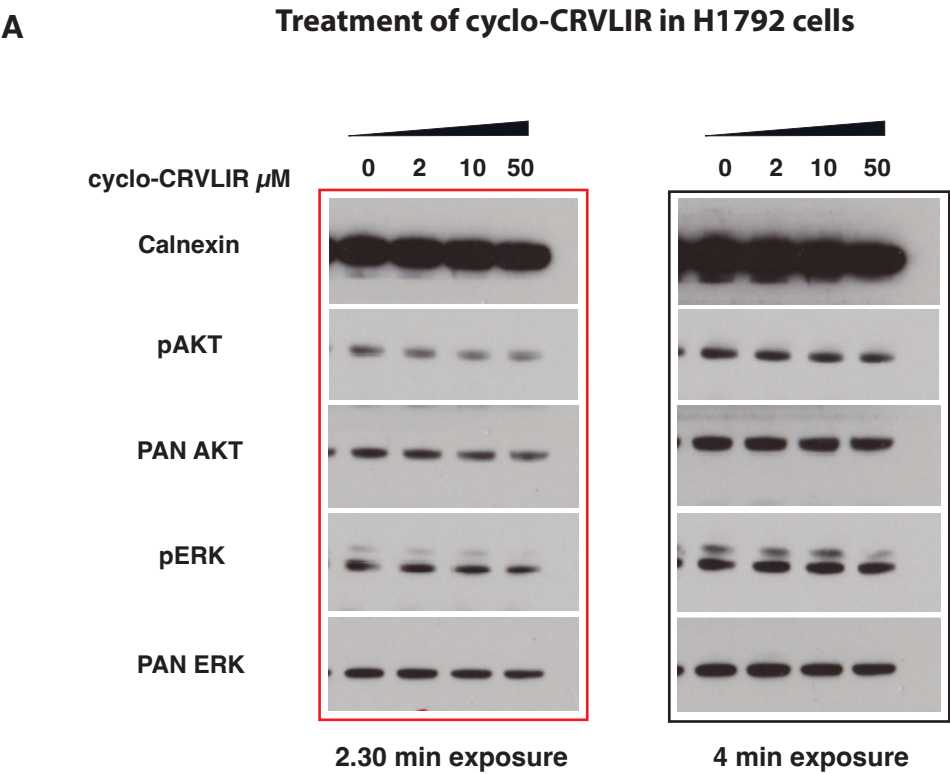

**B** Treatment of cyclo-CRVLIR in H1792 cells  
original uncropped membranes

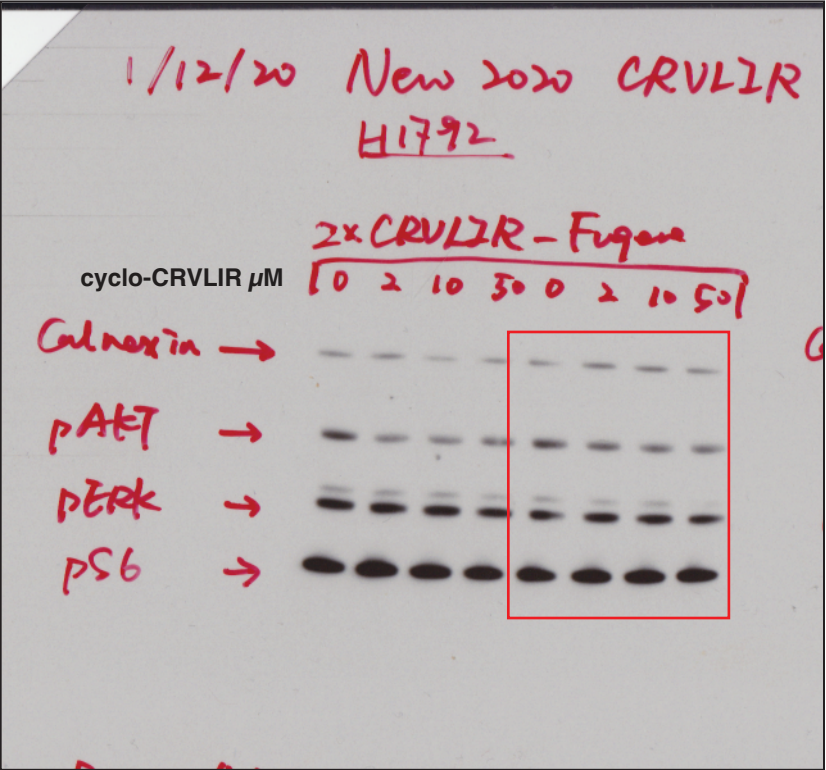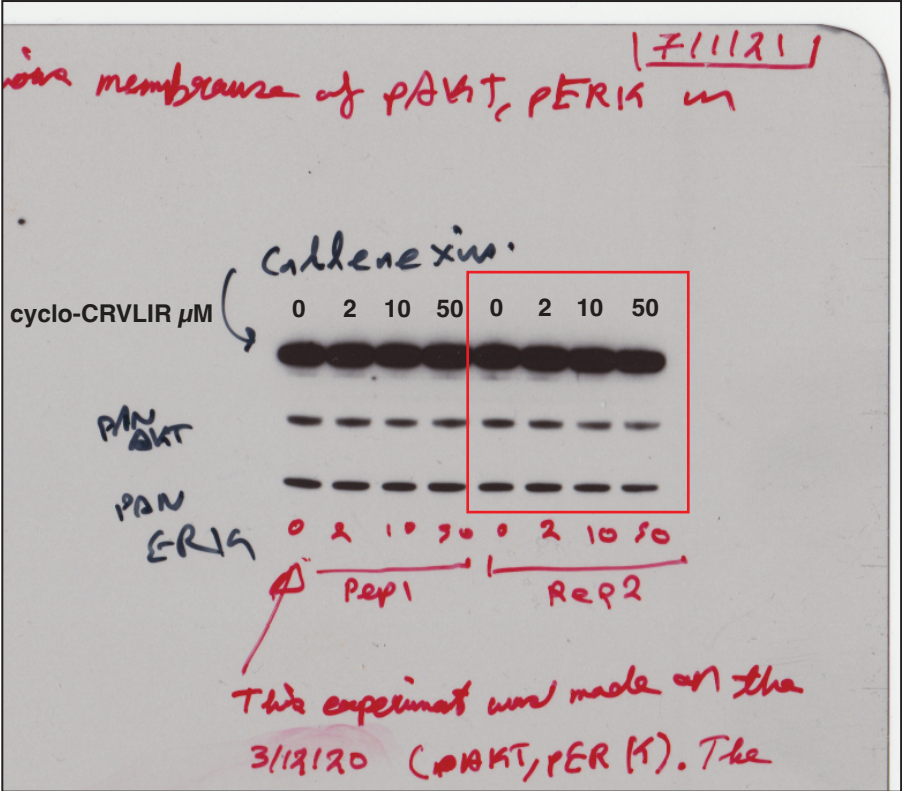

Supplement: Supplementary file 8 — Supplementary Information 8. [file 41598_2023_28756_MOESM8_ESM.pdf]
