# Supplementary material for: Characterisation of a cyclic peptide that binds to the RAS binding domain of phosphoinositide 3-kinase p110α
Source: Sci Rep. 2023 Feb 2;13:1889. doi: 10.1038/s41598-023-28756-0 (PMC9894841; doi:10.1038/s41598-023-28756-0)

Supplementary Figure 6

A Treatment of H1792 cells with cyclo-CRVLIR

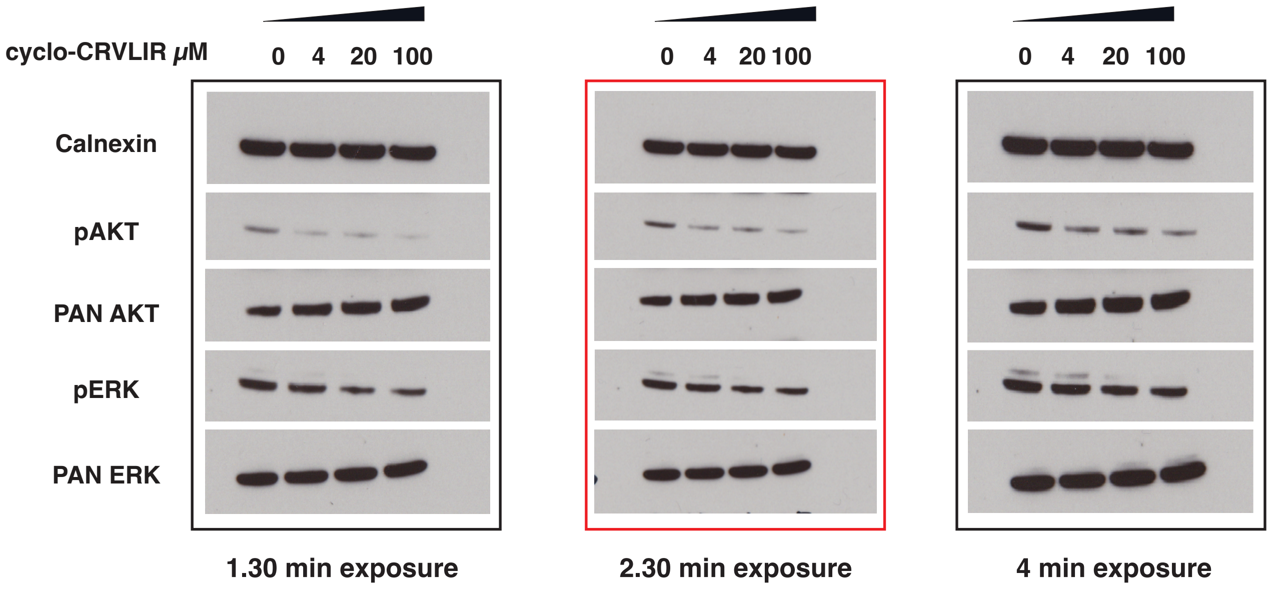

B Treatment of H1373 cells with cyclo-CRVLIR

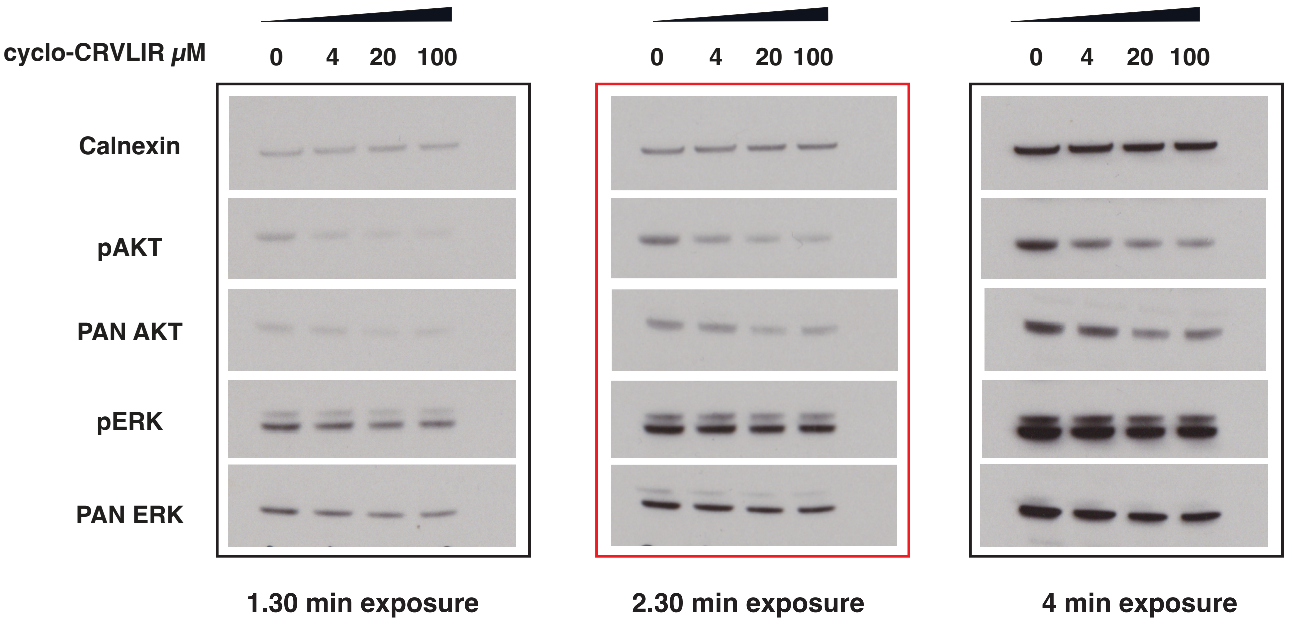

C Treatment of H1792 cells with cyclo-CRVLIR original uncropped membranes

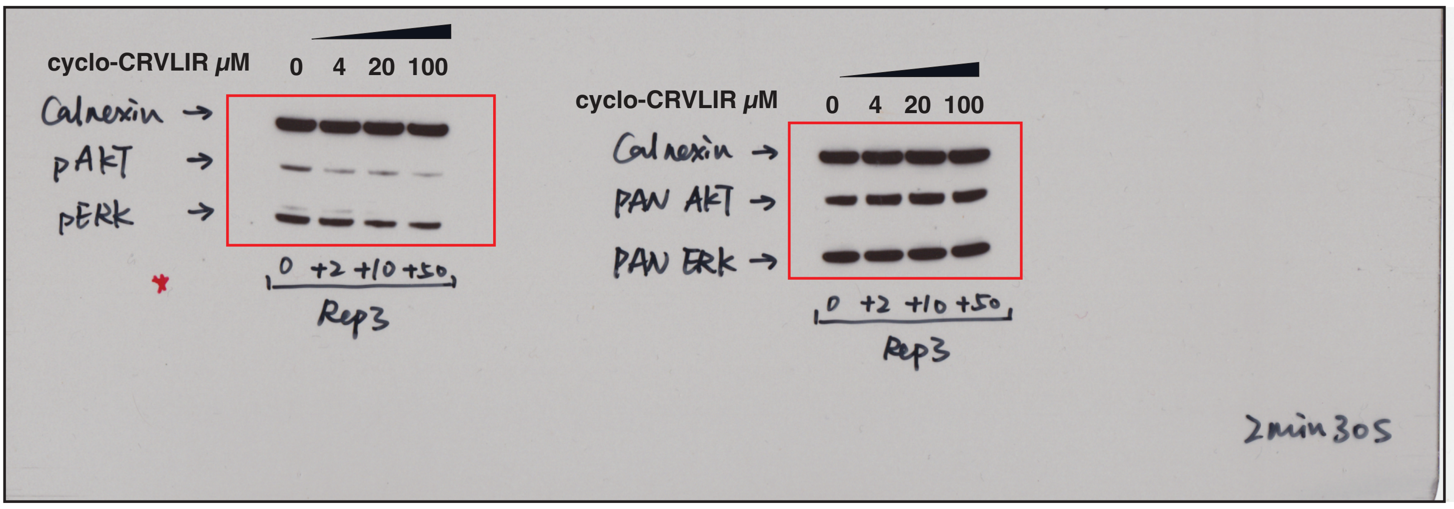

D Treatment of H1373 cells with cyclo-CRVLIR original uncropped membranes

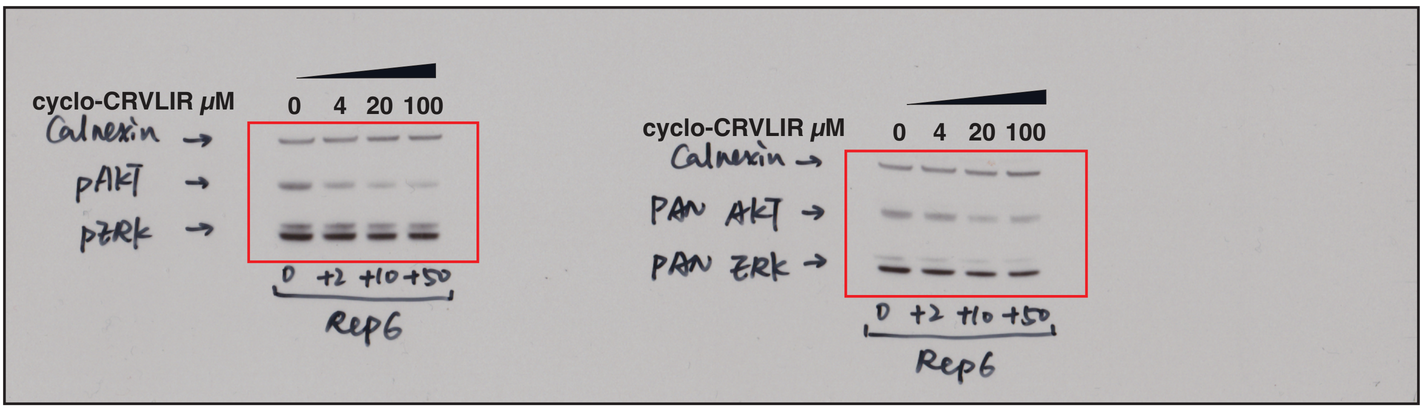

Supplement: Supplementary file 7 — Supplementary Information 7. [file 41598_2023_28756_MOESM7_ESM.pdf]
