# Supplementary material for: Characterisation of a cyclic peptide that binds to the RAS binding domain of phosphoinositide 3-kinase p110α
Source: Sci Rep. 2023 Feb 2;13:1889. doi: 10.1038/s41598-023-28756-0 (PMC9894841; doi:10.1038/s41598-023-28756-0)

Supplementary Figure 8

cyclo-CRVLAA 2nd derivatives in H1792 cancer cell line

A

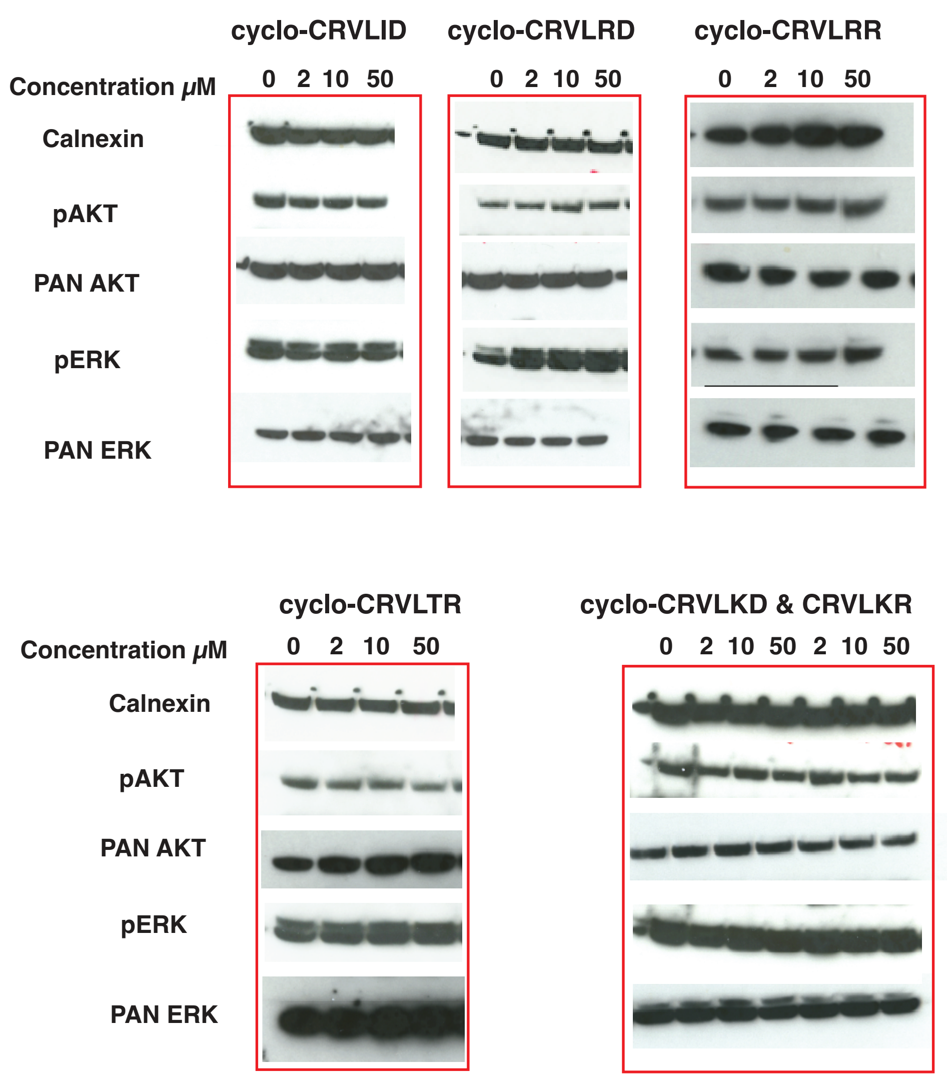

B

cyclo-CRVLAA 2nd derivatives in H1792 cancer cell line original uncropped membranes

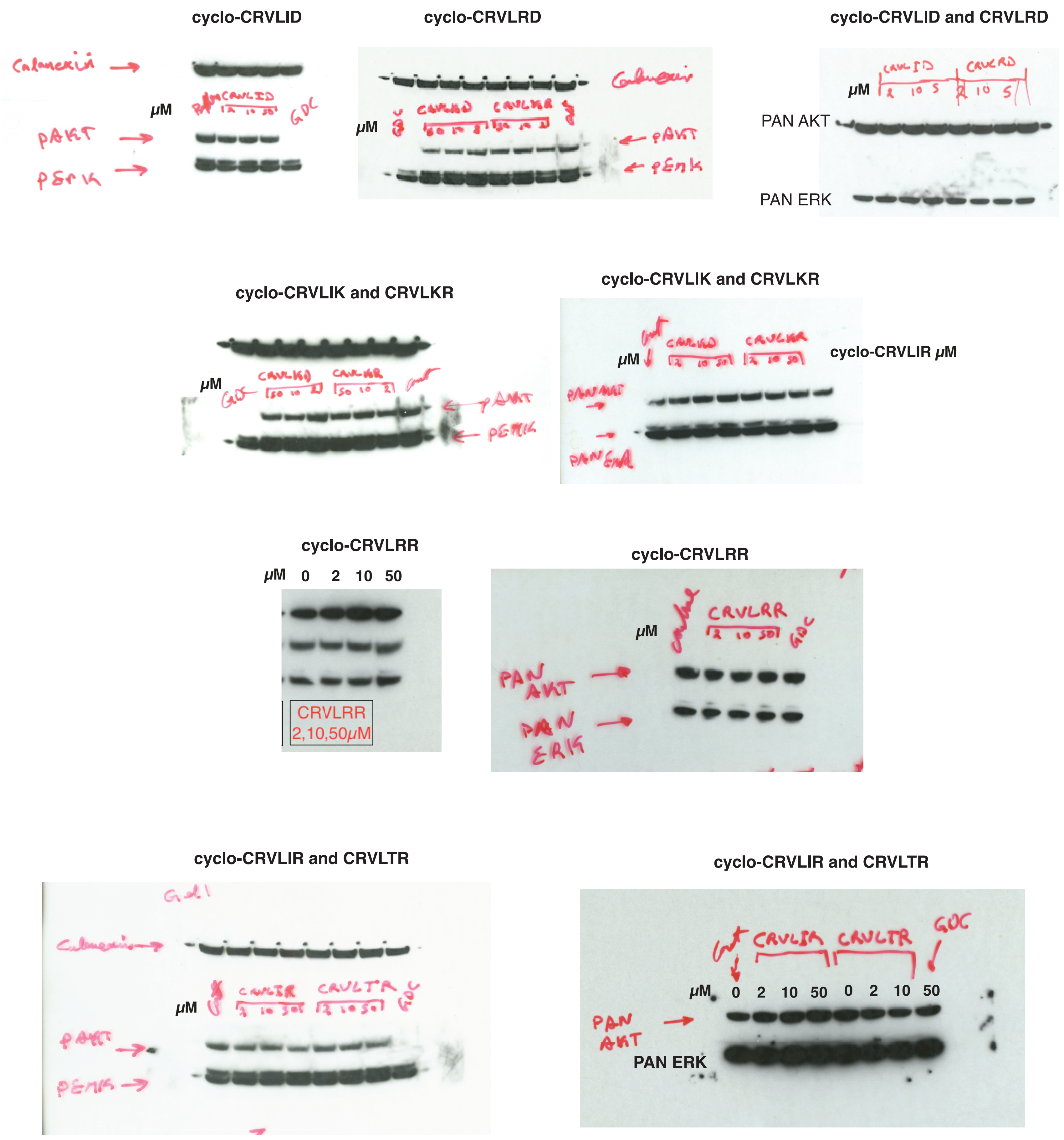

Supplement: Supplementary file 9 — Supplementary Information 9. [file 41598_2023_28756_MOESM9_ESM.pdf]
